# Supplementary material for: Assessing the inference of single-cell phylogenies and population dynamics from CRISPR lineage recordings
Source: PLoS Comput Biol. 2026 Jun 8;22(6):e1014370. doi: 10.1371/journal.pcbi.1014370 (PMC13268653; doi:10.1371/journal.pcbi.1014370)
Supplement: Attachment — Submitted filename: Rebuttal letter.pdf [file pcbi.1014370.s002.pdf]

Eidgenössische Technische Hochschule Zürich
Swiss Federal Institute of Technology Zurich

Julia Pilarski
Department of Biosystems Science and Engineering
Computational Evolution Group
julia.pilarski@bsse.ethz.ch

Basel, 2 April 2026

Dimitrios Vavylonis
Section Editor
PLOS Computational Biology

Re: Revision of manuscript "Assessing the inference of single-cell phylogenies and population dynamics from genetic lineage tracing data"

Dear Professor Vavylonis,

We thank you for the opportunity to submit a revised version of our manuscript. We also thank both reviewers for their comments and suggestions, which we believe have strengthened our work.

We have addressed the reviewer comments and have highlighted any changes in the manuscript. Our point-by-point responses to the reviewers are below (in blue).

Sincerely,

Julia Pilarski,
on behalf of all authors

Reviewer #1:

This manuscript performs several types of simulations of CRISPR-based recorders on lineage trees, does Bayesian inference of cell lineages, and compares the results to the simulated ground truth. The authors answer questions about what can be learned from these data using Bayesian methods.

The paper solidly delivers within its scope. The comparisons are appropriate, and the text and figures are very readable. I have not rerun the analysis, but what I can see in the GitHub repository inspires confidence.

> We thank the reviewer for their comments, and respond to each open point below. (The line numbers refer to the marked-up version of the manuscript.)

My main question is: are these results of interest for practitioners of these techniques? Would they use these results to guide experiments?

> In fact, this study emerged from questions raised by practitioners of the CRISPR lineage tracing techniques. Currently, we are actively collaborating with the Seattle Hub of Synthetic Biology, where studies such as ours and and also others are shaping the experimental design toward a lineage recorder mouse model. Especially questions around how many targets and what editing rate are needed, and whether sequential or non-sequential recording technologies are advantageous to estimate an accurate phylogeny and other dynamics come up repeatedly.
>
> In our simulations, we varied experimental parameters which are fundamental design choices in CRISPR lineage tracing (recorder type and capacity, editing rate and window). Experimentalists have substantial control over these, and our results are supporting their decision-making process. Especially having ballpark estimates for the required number of targets and the appropriate editing rate to reliably a) reconstruct the phylogeny and b) estimate cell population-dynamic parameters, such as division and differentiation rates for particular cell state hierarchies, for datasets with different number of cells, is crucial for designing experiments that generate biologically meaningful data. Our simulations provide insights on what information on cell development can be robustly inferred from different lineage recordings. Based on our findings, we now formulate practical recommendations for designing lineage tracing experiments in the future (L620-634).

Is there actually a discussion about whether to use sequential and non-sequential barcodes, or is everyone doing the former now?

> Non-sequential CRISPR–Cas9-based recorders, which accumulate indels at multiple independent target sites, were introduced earlier (e.g. see McKenna et al. 2016, Spanjaard et al. 2018 and others, around 2018). As a result, they dominate the literature, have been implemented in a broader range of biological systems, and have more experimental protocols and downstream analysis tools. Sequential recorders based on CRISPR prime editing, which write ordered insertions onto arrays of target sites, emerged more recently (around 2022, e.g. Choi et al. 2022). They are increasingly being adopted, but are not yet as widespread.
>
> We agree that practical implications of choosing between these two types of recorders should be more explicit. In L483-498, we added a paragraph to the discussion section describing how our study informs the debate on choosing between both recorder types based on the quantitative differences observed in our simulations.

These trees are small by barcode-editing standards: ~ 100 cells. Are practitioners happy with subsetting their data to that scale? Or do we know that the same results will hold with a much bigger tree?

Indeed, the throughput of CRISPR lineage tracing experiments is steadily increasing, and thousands of cells can now be edited and profiled in a single experiment. The Bayesian methods, however, currently do not allow for analyses on trees with more than approximately a thousand tips. The main reason is that the tree space grows super-exponentially with tree size, making exhaustive exploration infeasible.

Despite this unfavorable scaling, the Bayesian approach is exceptionally flexible and powerful in the amount of information that can be jointly inferred from relatively sparse data. In particular, the tree topology, branch lengths (in real time), and population-dynamic parameters (cell division, differentiation, and death rates) can be simultaneously estimated within a coherent probabilistic framework. As a result, analyses on small samples can still yield rich and biologically meaningful insights. Subsetting to hundreds of cells is therefore a pragmatic strategy, especially when the goal is detailed inference of temporal and dynamic processes rather than rough lineage reconstruction for all sampled cells.

We hypothesized how the posterior distributions will look like when the inference is performed on larger trees (as before L612-619 and L695-704). We have now added simulations on tenfold larger trees (with roughly ~1K tips) at the “baseline” (for both the non-sequential and sequential recorder) to test our hypotheses. We report detailed results in SI, Section S5. There, we also compare the runtime of inference on large vs. small trees, address current computational limitations of the methods, and discuss ongoing efforts in the field to improve their scalability.

Crucially, the additional results are consistent with our previous observations and the theory. Hence, we are confident that the qualitative trends identified in our simulations and described in the manuscript – in particular, the relative performance of phylogenetic and phylodynamic inference across scenarios – generalize to larger trees.

I know that estimation of death rates is an obsession of the phylodynamics community, but is this of interest for cell lineage folks as well?

The exact cell death rate might not be of highest priority, but the net growth rate (cell division rate β – cell death rate δ) of a cell population, and the turnover rate (relative rate of cell death vs. cell division δ/β) are key quantities of cell development. We now included the growth rate in our comparisons across scenarios (e.g. Figs. 2-4).

Moreover, cell death has been reported to be a coordinated process during embryogenesis (Saunders, 1966), hence, quantifying its occurrence is of interest to developmental biologists. There also seems to be interest in the regulatory mechanisms of cell death for treatment of disease. Prospectively, phylodynamic modelling could be used to pinpoint time periods or cell states with altered levels of cell death.

Are the simulations realistic enough to be able to shed light on these questions? An identical model is used for simulation and inference, and this model is quite idealized. A simple model makes sense for an inferential model, but I would be surprised if the real data is so clean. Don't these CRISPRs do strange things sometimes? Would it be interesting to put in some model violations for simulation? Is the Markov assumption built into the Birth-Death model appropriate?

We agree that incorporating major sources of noise in CRISPR lineage recordings would make our simulations more realistic. Therefore, in Section 2.5 (L381ff), we introduce simulations with target

silencing and sequencing dropout, for which the inferential models do not account for. For inference, we filtered alignments and cells to a subset of cells with maximally informative tapes/ targets, as currently done in practice (see Choi et al. 2022). Briefly, these analyses showed that population dynamic parameters were inferred with comparable accuracy from filtered recordings.

Further, we agree that cells exhibit rather non-Markovian dynamics in empirical systems – especially at early embryonic stages, cell divisions and deaths seem to occur after characteristic generation times rather than exponential waiting times, and the processes are not memoryless. Our simulations on “synchronous” trees were intended to cover an alternative extreme, i.e. almost deterministic, regular cell divisions and deaths, posing the highest discrepancy between the phylodynamic models used for simulation and inference. In this way, we assessed the (practical) appropriateness of the birth-death model (the current standard in single-cell phylogenetics) for inference in cell populations that evolved under non-Markovian dynamics. We modified the introductory paragraph (L97-106) to better motivate these simulations.

Relatedly, in L213 it would help to insert a little reminder of what a wRF of 0.2 would mean. Is this somehow normalized by tree lengths? Do the authors believe that they are able to recover at this level from real data?

The weighted Robinson-Foulds (wRF) distance measures dissimilarity between two phylogenetic trees by summing the differences in branch lengths of shared splits and adding the branch lengths of non-shared splits. Indeed, we reported wRF distances normalized by the sum of tree lengths, taking values between 0 and 1, where 0 indicates identical trees. Hence, the relatively low value of 0.2 means that most splits are the same, and that disagreements involve few splits, or short branches which contribute little weight, or both (now added to L243-250). Given the lack of ground-truth trees for empirical lineage recordings, it is difficult to evaluate tree recovery from real data. However, the results are consistent with independent simulations and the benchmark of tree reconstruction using the Bayesian editing models SciPhy and TiDeTree (cf. Figure 4 in Seidel, Zwaans et al., 2024).

The manuscript states "less accurate phylogeny reconstruction goes along with less certain estimation of phylodynamic parameters": is this actually established in the manuscript? Looking at, e.g., Fig 3, it's not obvious to me that this is true.

We agree that the trends shown initially in Figure 3 were subtle and did not directly show the association. We have hypothesized that less accurate phylogeny reconstruction, as measured by wRF distance, potentially means more errors and higher uncertainty in the branch lengths. Uncertainty in the branching times, in turn, propagates in increased uncertainty in the phylodynamic estimates (L326-332). We now plot these associations explicitly in Figs. 3C, S5 and S8.

Details:

Fig 2: the first plot: what are the lines and the points? Eg median and IQR?

Each point in this so-called “lollipop” chart shows the percentage of simulations, for which the true parameter has been recovered. The lines only serve visual comparability. We have clarified this in the figure legend.

The manuscript switches between "cell division rate" vs "birth rate", and it would be nice to use consistent terminology.

We now use the term “cell division rate” throughout the manuscript.

The synchronous simulations seemed so abiological until they were motivated late in the manuscript -- I suggest introducing that motivation earlier.

We put the motivation in the Introduction (L97-106).

L13 "can be displayed in": suggest "can be represented as"
L59 "’edits’": don't forget your TeX quotes! Should be `edits' in tex source.
L61 comma splice: remove comma before "as they are passed"
L117 "the terms and 'phylogeny'": redundant "and"
L189 "extend": should be "extent"
L198 "birth-date": should be "birth-death"
L201 "misspecificaton": should be "misspecification"
L224 "differences to": should be "differences from"
L227 "Fig. B3 and B4": should be "Figs. B3 and B4"
L564: did you mean to say "that a few"? That would mean the opposite.

We fixed the errors.

Reviewer #2:

This manuscript presents a simulation study evaluating a Bayesian framework for inferring phylodynamic parameters, extending beyond cell phylogenies, in lineage tracing experiments using barcodes. The topic is timely and relevant, and the study applies appropriate methodology, producing results of potential value to researchers working with similar data. The core contribution is interesting and generally well executed.

However, the substantial computational demands might have limited the exploration of the parameter space. This constraint may reduce the representativeness of the findings relative to real experimental scenarios. Addressing this limitation and more clearly situating the work within existing literature would significantly strengthen the study.

> We thank the reviewer for his insightful comments. In the revised manuscript, we have elaborated on the limitations of our approach and put it stronger in the context of existing work. We have also performed additional simulations and analyses which hopefully strengthen the study. Below, we respond to each comment in more detail.

-- Major comments –

1. Relation to Existing Work
1.1 Simulations similar in spirit have already been presented in TiDeTree [16], GABI [17], and SciPhy [18], and related methodologies for phylodynamic inference (e.g., scPhyloX: https://www.sciencedirect.com/science/article/abs/pii/S2405471225000778) are available. Explicitly clarifying the novelty of the present study—methodological, conceptual, or empirical—would help readers place this contribution in the broader context of lineage tracing and Bayesian phylodynamics.

> The simulations in TiDeTree, GABI and SciPhy publications have been conducted to validate the editing models implemented as BEAST2 packages (and similarly in scPhyloX, they have served method validation and benchmark). Here, we address the question: *How much information can we extract from lineage tracing data when analyzing it with the currently available computational methods*? We operate in the Bayesian framework, which allows us to couple the editing models to existing phylodynamic models for inference and capture uncertainty in the inference outputs.
>
> Our study highlights that phylogenetic reconstruction from lineage recordings is not perfect, even with full agreement between the simulation and inference models for the CRISPR barcode editing processes. Certain features of the recorders – in particular, their limited capacity – constrain the signal. By simulating lineage recordings at various experimental designs, we can investigate their downstream effects on tree reconstruction and estimation of population-dynamic parameters.
>
> Note that novel techniques for lineage tracing are being continuously developed to push the capacity limits and improve the resolution of the lineage recordings. In particular, technologies that employ prime editors and sequential editing aim to increase the amount of information that can be readout from cell barcodes. Our study provides the first (to our knowledge) quantitative comparison of the recorders and delineates how much improvement can be achieved.
>
> Additionally, previous studies have used the same population-dynamic models for tree simulation and inference. In particular, the models assumed exponentially distributed waiting times for cell divisions and deaths. In our study, we assess the effects of phylodynamic model misspecification by simulating trees under regular, synchronous cell divisions and deaths (an alternative “extreme”).

Furthermore, our study showcases how the CRISPR editing models can be combined with already existing phylodynamic models (so far mainly applied in epidemiology) in the Bayesian framework to infer cell type-specific division, death, and cell type transition dynamics, and reconstruct cell differentiation trajectories along lineage trees.

We highlighted the novelty of our study at several places in the revised manuscript, e.g. L82-87, L97-106, L107-122, L568-581.

2. Simulation scope and realism

2.1 The baseline simulation uses only 20 trees per condition, and the non-baseline conditions also involve relatively few trees and scenarios. The number of simulated cells and targets is considerably lower than typical real-world lineage tracing datasets. Scaling up selected simulations could provide more realistic variability.

Indeed, we simulated 100 trees per experimental design, specifically 20 trees for each of the five tree generating processes. Given the high computational cost per inference, we decided not to add more replicates, as we see consistent inference outputs per condition, e.g. similar posterior distributions of phylodynamic parameters despite variable tree statistics (cf. for example Fig. S1).

Further, we find the number of targets (varying from 5 to 40 independent targets, or 1x20 to 40x5 tapes with sequential targets) to be representative for empirical lineage tracing datasets, comparing e.g. to barcodes recovered in recordings in zebrafish with LINNAEUS (16-32) and ScarTrace (8), in mice with DARLIN (3x10) and hgRNAs (60), in HEK283T cells (13x5) and gastruloids (8x6) with DNA Typewriter.

We acknowledge that the number of cells in our simulations is relatively low. In the Bayesian framework, however, we are limited to analyses with a few hundreds to a thousand cells. We scaled up baseline simulations to tenfold larger trees (with 200-2000 cells) to assess whether inference from such larger samples is feasible and how the tree size affects the inference performance. We report and discuss the results in detail in Supplementary Material, Section S5. Briefly, we find that our results on smaller trees generalise to the larger tree inferences.

2.2 In the current simulations, most cell divisions occur later in the timelines. Please clarify whether this reflects typical patterns in barcoding experiments or is specific to the chosen simulation settings.

CRISPR lineage recorders are mainly employed to study early embryonic development in model organisms (e.g. zebrafish and mice), or other cell populations (e.g. gastruloids) that originate from a single or few cells with initially unedited targets. These cell populations are growing exponentially. Thus, considering the whole population, the total number of cell divisions increases with time. In cancer mouse models, usually cells from a tumor line are injected into the blood stream and then their behaviour is analysed. There we would also expect few cells to initialise a tumor.

2.3 The editing rate is fixed at 0.05 in the baseline scenario. Is this representative of actual experiments? Given the flexibility of the simulation framework, exploring a range of editing rates and potentially identifying optimal rates for sequential vs. non-sequential recording schemes could provide practical insights for empirical researchers.

The editing rate is defined as the number of edits per active target per time unit. As edits are irreversible, the number of active targets decreases over time. So 0.05 given 20 targets and 40 time units translates to initially 1 edit per time unit on any of the 20 targets, and less at later time points.

The editing rates of 0.01–0.45 used for simulations fall in the range of previously inferred rates (e.g. 0.1–0.3 in HEK cells and 0.05–0.6 in gastruloids). More importantly, the non-sequential/ sequential recorder saturation of ~85/40% at the editing rate of 0.05 is realistic in actual experiments.

Our study highlights that the editing rate should be tuned relative to the number of targets and duration of the experiment (i.e. the time between origin of the developmental process and sampling cells for sequencing). We investigated three editing rate regimes resulting in different levels of saturation of the distinct lineage recorders (cf. Table S2). While inference performance clearly dropped when edits were very sparse or the recorders oversaturated, we did not observe substantial differences when the editing outcomes across targets and cells were sufficiently diverse (particularly, comparing the baseline and near-optimal editing rate – resulting in ~90% saturation – for the sequential recorder, L333-344). Based on these findings, we recommend performing forward simulations, which is fast and computationally inexpensive, prior to empirical experiments to fine-tune the editing rate to the chosen recorder type, its capacity and recording duration (L620-634).

2.4 Key real-world complexities, such as observational noise, have been omitted. Incorporating noise could help assess robustness. Similarly, varying cell numbers would allow evaluation of the method's behavior when applied to realistic experimental datasets, where model misspecification is inevitable.

Indeed. We have added simulations incorporating two major sources of noise in CRISPR lineage recordings, i.e. target silencing and sequencing dropout. We have then performed a filtering step, as is usually done in real-world data analysis (e.g. see Choi et al. 2022) and assessed its impact on the inference. We present the results in Section 2.5 (L381ff). Briefly, these analyses showed that population dynamic parameters were inferred with comparable accuracy from filtered recordings.

2.5 Biological heterogeneity—such as birth–death rate differences caused by driver mutations in cancer—could be explored via multitype BD models. An unordered transition model among clone types might be suitable for capturing competing clonal dynamics.

We have added a paragraph in the Discussion (L568-581), describing how the multi-type scenarios in our simulation study – mimicking cell type transitions due to differentiation, e.g. from stem cell to more specialized types – could be generalized to disease conditions, particularly to the emergence of clones in cancer. We also refer to the recent application of structured phylodynamic models to single-cell whole-genome sequencing data from colorectal cancer to infer malignant cell population dynamics over time (Alves et al., 2025).

However, our study focuses on the properties of different CRISPR-based lineage recorders. Such engineered systems cannot be introduced into patients and are therefore unsuitable for studying tumor dynamics in clinically relevant settings. In contrast, cancer cells typically exhibit substantial genomic variation that can be leveraged for phylogenetic and phylodynamic analysis.

We further note that in other fields (epidemiology, macroevolution), unordered transitions between types were shown to be inferred under multi-type BD models – for example, viral transmissions across geographic compartments (e.g. Kühnert et al., 2016). Such transitions can be, in principle, also inferred when types represent cell states or clones, given that the single-cell phylogenetic trees can be reliably estimated in the framework. Structured phylodynamic models can explicitly account for type-specific birth and death rates, which has been shown to be critical for accurately recovering transition rates between subpopulations (Seidel et al. 2024). This property is equally relevant in the cellular setting, where different cell types or clones may proliferate and die at different rates.

More broadly, we agree that inferring transition graphs, beyond estimating transition rates, is of considerable interest in both developmental biology and cancer. As methods scale to accommodate

larger numbers of cells, more cell types, and increasingly complex transition scenarios, we anticipate exciting applications of Bayesian phylodynamic inference to single-cell lineage tracing datasets.

3. Results exploration

3.1 The manuscript notes overestimation of the transition rate to type 2 but leaves this for future work. A brief, even preliminary, analysis of possible causes would enhance the current study.

We inspected the posterior set of trees for which the transition rate was overestimated (cf. Fig. S15). The overestimation is consistent with mapping transitions to type 2 earlier in the tree-generating process. We explain this particular model behavior in more detail in L443-457.

3.2 The timing of cell state transitions could be of much interest to experimentalists. Is this estimable with the present simulations? If so, it would be worth reporting.

In fact, the stochastic mapping algorithm (implemented in the BDMM-Prime package) “colors” posterior trees, i.e. maps type transitions onto the inferred phylogenies (L430-433). In Fig. S14, we show examples of such posterior sets of “colored” trees together with the summary trees (MCC trees with type probabilities at internal nodes) and the corresponding true trees.

Consequently, the timings of cell type transitions can be estimated from the simulated recordings and compared to the ground-truth. In Fig. S16, we show that the following transition statistics could be well recovered across simulations:

- number of transitions to each type
- time of first transition to each type
- total time spent in each type (corresponding to the sum of branch lengths per type)

(cf. L465-468)

4. Discussion and applicability

4.1 It would be helpful to expand on why sequential editing did not improve inference of phylodynamic parameters.

We have hypothesized that sequential editing significantly improved inference of the tree topology, but not the branch lengths. The weighted Robinson-Foulds (wRF) distance, however, measures both. We have now computed different measures for topology only (RF distance, Shared Phylogenetic Information), and branch lengths only (Wasserstein and Kolmogorov-Smirnov distance between the true and inferred branch length distribution). While topological improvements are consistently significant, sequential editing seems to only slightly improve the estimation of branch lengths, and consequently, the phylodynamic parameters (L371-380).

4.2 Computational time is a nontrivial concern for real-world applicability. The manuscript should more explicitly address whether the method can be applied to typical experimental datasets in its current form, and outline possible strategies to reduce runtime (e.g., algorithmic optimization, parallelization, approximations). This is particularly important given the stated aim of incorporating more complex models in the future.

Indeed, the computational runtime is currently the major bottleneck in Bayesian phylogenetic and phylodynamic analysis of single-cell data. We have addressed the challenges, and outlined strategies to reduce runtime and improve scalability of the methods in Supplementary Material, Section S5.3.

-- Minor comments --

5.1. The manuscript uses “apoptosis” interchangeably with “cell death.” Apoptosis is only one mode of cell death; necrosis and autophagy are others. Using “cell death” as a general term would be more accurate.

Agreed. We have changed the instances to “cell death”.

5.2 The phrase “genetic lineage tracing” may be overbroad, as it can also refer to phylogenetic reconstruction from single-nucleotide variants. In this context, the term “barcode lineage tracing” may be clearer.

Agreed. We have modified the title to “*Assessing the inference of single-cell phylogenies and population dynamics from CRISPR lineage recordings”,* and use the term “CRISPR” instead of “genetic” in the text.
