# Supplementary material for: Assessing the inference of single-cell phylogenies and population dynamics from CRISPR lineage recordings
Source: PLoS Comput Biol. 2026 Jun 8;22(6):e1014370. doi: 10.1371/journal.pcbi.1014370 (PMC13268653; doi:10.1371/journal.pcbi.1014370)
Supplement: Attachment — Submitted filename: Rebuttal letter - minor revision.pdf [file pcbi.1014370.s003.pdf]

Eidgenössische Technische Hochschule Zürich
Swiss Federal Institute of Technology Zurich

Julia Pilarski
Department of Biosystems Science and Engineering
Computational Evolution Group
julia.pilarski@bsse.ethz.ch

Basel, 21 May 2026

Dimitrios Vavylonis
Section Editor
PLOS Computational Biology

Re: Revision of manuscript "Assessing the inference of single-cell phylogenies and population dynamics from CRISPR lineage recordings"

Dear Professor Vavylonis,

Thank you for the positive evaluation of our manuscript. We have addressed the remaining minor comment in the revision. Following the reviewer's suggestion, we have emphasized in the text that non-sequential recorders remain actively utilized and optimized, and provided more recent references to support this statement.

Sincerely,

Julia Pilarski,
on behalf of all authors
