# Supplementary material for: Assessing the inference of single-cell phylogenies and population dynamics from CRISPR lineage recordings
Source: PLoS Comput Biol. 2026 Jun 8;22(6):e1014370. doi: 10.1371/journal.pcbi.1014370 (PMC13268653; doi:10.1371/journal.pcbi.1014370)
Supplement: Peer review history

# Peer review: Decision Letter 0

**DOI:** 10.1371/journal.pcbi.1014370.r001

**Authors:** Dimitrios Vavylonis

26 Nov 2025

PCOMPBIOL-D-25-01344

Assessing the inference of single-cell phylogenies and population dynamics from genetic lineage tracing data

PLOS Computational Biology

Dear Dr. Pilarski,

Thank you for submitting your manuscript to PLOS Computational Biology. We apologize for the delay, which was due to a previous editor becoming unavailable. After careful consideration, we feel that it has merit but does not fully meet PLOS Computational Biology’s publication criteria as it currently stands. Reviewers expressed major concerns, especially about the demonstrated usefulness of the results for practitioners and their applicability to real-world datasets. Therefore, we invite you to submit a revised version of the manuscript that addresses the points raised during the review process.

Please submit your revised manuscript by Jan 26 2026 11:59PM. If you will need more time than this to complete your revisions, please reply to this message or contact the journal office at ploscompbiol@plos.org. When you’re ready to submit your revision, log on to https://www.editorialmanager.com/pcompbiol/ and select the ‘Submissions Needing Revision’ folder to locate your manuscript file.

Please include the following items when submitting your revised manuscript:

* A rebuttal letter that responds to each point raised by the editor and reviewer(s). You should upload this letter as a separate file labeled ‘Response to Reviewers’. This file does not need to include responses to formatting updates and technical items listed in the ‘Journal Requirements’ section below.

* A marked-up copy of your manuscript that highlights changes made to the original version. You should upload this as a separate file labeled ‘Revised Manuscript with Track Changes’.

* An unmarked version of your revised paper without tracked changes. You should upload this as a separate file labeled ‘Manuscript’.

If you would like to make changes to your financial disclosure, competing interests statement, or data availability statement, please make these updates within the submission form at the time of resubmission. Guidelines for resubmitting your figure files are available below the reviewer comments at the end of this letter

We look forward to receiving your revised manuscript.

Kind regards,

Dimitrios Vavylonis

Section Editor

PLOS Computational Biology

**Journal Requirements:**

1) Please ensure that the CRediT author contributions listed for every co-author are completed accurately and in full.

At this stage, the following Authors/Authors require contributions: Julia Pilarski, Tanja Stadler, and Sophie Seidel. Please ensure that the full contributions of each author are acknowledged in the "Add/Edit/Remove Authors" section of our submission form.

The list of CRediT author contributions may be found here: https://journals.plos.org/ploscompbiol/s/authorship#loc-author-contributions

2) We ask that a manuscript source file is provided at Revision. Please upload your manuscript file as a .doc, .docx, .rtf or .tex. If you are providing a .tex file, please upload it under the item type u2018LaTeX Source Fileu2019 and leave your .pdf version as the item type u2018Manuscriptu2019.

3) Please upload all main figures as separate Figure files in .tif or .eps format. For more information about how to convert and format your figure files please see our guidelines:

https://journals.plos.org/ploscompbiol/s/figures

4) We notice that your supplementary Figures, and Table are included in the manuscript file. Please remove them and upload them with the file type ‘Supporting Information’. Please ensure that each Supporting Information file has a legend listed in the manuscript after the references list.

5) Some material included in your submission may be copyrighted. According to PLOSu2019s copyright policy, authors who use figures or other material (e.g., graphics, clipart, maps) from another author or copyright holder must demonstrate or obtain permission to publish this material under the Creative Commons Attribution 4.0 International (CC BY 4.0) License used by PLOS journals. Please closely review the details of PLOSu2019s copyright requirements here: PLOS Licenses and Copyright. If you need to request permissions from a copyright holder, you may use PLOS’s Copyright Content Permission form.

Please respond directly to this email and provide any known details concerning your material’s license terms and permissions required for reuse, even if you have not yet obtained copyright permissions or are unsure of your material’s copyright compatibility. Once you have responded and addressed all other outstanding technical requirements, you may resubmit your manuscript within Editorial Manager.

Potential Copyright Issues:

i) Figures 1, and 5. Please confirm whether you drew the images / clip-art within the figure panels by hand. If you did not draw the images, please provide (a) a link to the source of the images or icons and their license / terms of use; or (b) written permission from the copyright holder to publish the images or icons under our CC BY 4.0 license. Alternatively, you may replace the images with open source alternatives. See these open source resources you may use to replace images / clip-art:

- https://commons.wikimedia.org

- https://openclipart.org/.

6) Please amend your detailed Financial Disclosure statement. This is published with the article. It must therefore be completed in full sentences and contain the exact wording you wish to be published.

1) State the initials, alongside each funding source, of each author to receive each grant. For example: "This work was supported by the National Institutes of Health (####### to AM; ###### to CJ) and the National Science Foundation (###### to AM)."

7) Please ensure that the funders and grant numbers match between the Financial Disclosure field and the Funding Information tab in your submission form. Note that the funders must be provided in the same order in both places as well. Currently, "ETH Zürich" is missing from the Funding Information tab .

Note: If the reviewer comments include a recommendation to cite specific previously published works, please review and evaluate these publications to determine whether they are relevant and should be cited. There is no requirement to cite these works unless the editor has indicated otherwise.

**Reviewers’ comments:**

Reviewer’s Responses to Questions

Reviewer #1: This manuscript performs several types of simulations of CRISPR-based recorders on lineage trees, does Bayesian inference of cell lineages, and compares the results to the simulated ground truth. The authors answer questions about what can be learned from these data using Bayesian methods.

The paper solidly delivers within its scope. The comparisons are appropriate, and the text and figures are very readable. I have not rerun the analysis, but what I can see in the GitHub repository inspires confidence.

My main question is: are these results of interest for practitioners of these techniques? Would they use these results to guide experiments?

* Is there actually a discussion about whether to use sequential and non-sequential barcodes, or is everyone doing the former now?

* These trees are small by barcode-editing standards: ~ 100 cells. Are practitioners happy with subsetting their data to that scale? Or do we know that the same results will hold with a much bigger tree?

* I know that estimation of death rates is an obsession of the phylodynamics community, but is this of interest for cell lineage folks as well?

Are the simulations realistic enough to be able to shed light on these questions? An identical model is used for simulation and inference, and this model is quite idealized. A simple model makes sense for an inferential model, but I would be surprised if the real data is so clean. Don’t these CRISPRs do strange things sometimes? Would it be interesting to put in some model violations for simulation? Is the Markov assumption built into the Birth-Death model appropriate?

Relatedly, in L213 it would help to insert a little reminder of what a wRF of 0.2 would mean. Is this somehow normalized by tree lengths? Do the authors believe that they are able to recover at this level from real data?

The manuscript states "less accurate phylogeny reconstruction goes along with less certain estimation of phylodynamic parameters": is this actually established in the manuscript? Looking at, e.g., Fig 3, it’s not obvious to me that this is true.

Details:

Fig 2: the first plot: what are the lines and the points? Eg median and IQR?

The manuscript switches between "cell division rate" vs "birth rate", and it would be nice to use consistent terminology.

The synchronous simulations seemed so abiological until they were motivated late in the manuscript-- I suggest introducing that motivation earlier.

L13 "can be displayed in": suggest "can be represented as"

L59 "’edits’": don’t forget your TeX quotes! Should be `edits’ in tex source.

L61 comma splice: remove comma before "as they are passed"

L117 "the terms and ‘phylogeny’": redundant "and"

L189 "extend": should be "extent"

L198 "birth-date": should be "birth-death"

L201 "misspecificaton": should be "misspecification"

L224 "differences to": should be "differences from"

L227 "Fig. B3 and B4": should be "Figs. B3 and B4"

L564: did you mean to say "that a few"? That would mean the opposite.

Reviewer #2: 25-PCOMPBIOL-D-25-01344-review

Assessing the inference of single-cell phylogenies and population dynamics from genetic lineage tracing data

Pilarski et al.

---

This manuscript presents a simulation study evaluating a Bayesian framework for inferring phylodynamic parameters, extending beyond cell phylogenies, in lineage tracing experiments using barcodes. The topic is timely and relevant, and the study applies appropriate methodology, producing results of potential value to researchers working with similar data. The core contribution is interesting and generally well executed.

However, the substantial computational demands might have limited the exploration of the parameter space. This constraint may reduce the representativeness of the findings relative to real experimental scenarios. Addressing this limitation and more clearly situating the work within existing literature would significantly strengthen the study.

-- Major comments --

1. Relation to Existing Work

1.1 Simulations similar in spirit have already been presented in TiDeTree [16], GABI [17], and SciPhy [18], and related methodologies for phylodynamic inference (e.g., scPhyloX: https://www.sciencedirect.com/science/article/abs/pii/S2405471225000778) are available. Explicitly clarifying the novelty of the present study—methodological, conceptual, or empirical—would help readers place this contribution in the broader context of lineage tracing and Bayesian phylodynamics.

2. Simulation scope and realism

2.1 The baseline simulation uses only 20 trees per condition, and the non-baseline conditions also involve relatively few trees and scenarios. The number of simulated cells and targets is considerably lower than typical real-world lineage tracing datasets. Scaling up selected simulations could provide more realistic variability.

2.2. In the current simulations, most cell divisions occur later in the timelines. Please clarify whether this reflects typical patterns in barcoding experiments or is specific to the chosen simulation settings.

2.3 The editing rate is fixed at 0.05 in the baseline scenario. Is this representative of actual experiments? Given the flexibility of the simulation framework, exploring a range of editing rates and potentially identifying optimal rates for sequential vs. non-sequential recording schemes could provide practical insights for empirical researchers.

2.4 Key real-world complexities, such as observational noise, have been omitted. Incorporating noise could help assess robustness. Similarly, varying cell numbers would allow evaluation of the method’s behavior when applied to realistic experimental datasets, where model misspecification is inevitable.

2.5 Biological heterogeneity—such as birth–death rate differences caused by driver mutations in cancer—could be explored via multitype BD models. An unordered transition model among clone types might be suitable for capturing competing clonal dynamics.

3. Results exploration

3.1 The manuscript notes overestimation of the transition rate to type 2 but leaves this for future work. A brief, even preliminary, analysis of possible causes would enhance the current study.

3.2 The timing of cell state transitions could be of much interest to experimentalists. Is this estimable with the present simulations? If so, it would be worth reporting.

4. Discussion and applicability

4.1 It would be helpful to expand on why sequential editing did not improve inference of phylodynamic parameters.

4.2 Computational time is a nontrivial concern for real-world applicability. The manuscript should more explicitly address whether the method can be applied to typical experimental datasets in its current form, and outline possible strategies to reduce runtime (e.g., algorithmic optimization, parallelization, approximations). This is particularly important given the stated aim of incorporating more complex models in the future.

-- Minor comments --

5.1. The manuscript uses “apoptosis” interchangeably with “cell death.” Apoptosis is only one mode of cell death; necrosis and autophagy are others. Using “cell death” as a general term would be more accurate.

5.2 The phrase “genetic lineage tracing” may be overbroad, as it can also refer to phylogenetic reconstruction from single-nucleotide variants. In this context, the term “barcode lineage tracing” may be clearer.

Overall, this is a valuable and timely contribution, and with the suggested refinements, it has the potential to make a substantial impact in the field. Congratulations to the authors on their solid work.

David Posada

**********

**Have the authors made all data and (if applicable) computational code underlying the findings in their manuscript fully available?**

The PLOS Data policy requires authors to make all data and code underlying the findings described in their manuscript fully available without restriction, with rare exception (please refer to the Data Availability Statement in the manuscript PDF file). The data and code should be provided as part of the manuscript or its supporting information, or deposited to a public repository. For example, in addition to summary statistics, the data points behind means, medians and variance measures should be available. If there are restrictions on publicly sharing data or code —e.g. participant privacy or use of data from a third party—those must be specified.

Reviewer #1: Yes

Reviewer #2: Yes

**********

PLOS authors have the option to publish the peer review history of their article (what does this mean?). If published, this will include your full peer review and any attached files.

If you choose “no”, your identity will remain anonymous but your review may still be made public.

**Do you want your identity to be public for this peer review?**    For information about this choice, including consent withdrawal, please see our Privacy Policy.

Reviewer #1: No

Reviewer #2: **Yes:**    David Posada

[NOTE: If reviewer comments were submitted as an attachment file, they will be attached to this email and accessible via the submission site. Please log into your account, locate the manuscript record, and check for the action link "View Attachments". If this link does not appear, there are no attachment files.]

**Figure resubmission:**

While revising your submission, we strongly recommend that you use PLOS’s NAAS tool (https://ngplosjournals.pagemajik.ai/artanalysis) to test your figure files. NAAS can convert your figure files to the TIFF file type and meet basic requirements (such as print size, resolution), or provide you with a report on issues that do not meet our requirements and that NAAS cannot fix.

After uploading your figures to PLOS’s NAAS tool - https://ngplosjournals.pagemajik.ai/artanalysis, NAAS will process the files provided and display the results in the "Uploaded Files" section of the page as the processing is complete. If the uploaded figures meet our requirements (or NAAS is able to fix the files to meet our requirements), the figure will be marked as "fixed" above. If NAAS is unable to fix the files, a red "failed" label will appear above. When NAAS has confirmed that the figure files meet our requirements, please download the file via the download option, and include these NAAS processed figure files when submitting your revised manuscript.

**Reproducibility:**

To enhance the reproducibility of your results, we recommend that authors of applicable studies deposit laboratory protocols in protocols.io, where a protocol can be assigned its own identifier (DOI) such that it can be cited independently in the future. Additionally, PLOS ONE offers an option to publish peer-reviewed clinical study protocols. Read more information on sharing protocols at https://plos.org/protocols?utm_medium=editorial-email&utm_source=authorletters&utm_campaign=protocols

# Author comment: Author response to Decision Letter 1

**DOI:** 10.1371/journal.pcbi.1014370.r002

7 Apr 2026

Attachment Submitted filename: Rebuttal letter.pdf

# Peer review: Decision Letter 1

**DOI:** 10.1371/journal.pcbi.1014370.r003

**Authors:** Dimitrios Vavylonis

12 May 2026

PCOMPBIOL-D-25-01344R1

Assessing the inference of single-cell phylogenies and population dynamics from CRISPR lineage recordings

PLOS Computational Biology

Dear Dr. Pilarski,

Thank you for submitting your manuscript to PLOS Computational Biology. We are happy to let you know that the reviewers were satisfied with the revisions and your manuscript will be accepted, pending addressing a very minor comment by one of the reviewers.

Please submit your revised manuscript by Jul 12 2026 11:59PM. If you will need more time than this to complete your revisions, please reply to this message or contact the journal office at ploscompbiol@plos.org. When you’re ready to submit your revision, log on to https://www.editorialmanager.com/pcompbiol/ and select the ‘Submissions Needing Revision’ folder to locate your manuscript file.

Please include the following items when submitting your revised manuscript:

* A letter that responds to each point raised by the editor and reviewer(s). You should upload this letter as a separate file labeled ‘Response to Reviewers’. This file does not need to include responses to formatting updates and technical items listed in the ‘Journal Requirements’ section below.

* A marked-up copy of your manuscript that highlights changes made to the original version. You should upload this as a separate file labeled ‘Revised Manuscript with Track Changes’.

* An unmarked version of your revised paper without tracked changes. You should upload this as a separate file labeled ‘Manuscript’.

If you would like to make changes to your financial disclosure, competing interests statement, or data availability statement, please make these updates within the submission form at the time of resubmission. Guidelines for resubmitting your figure files are available below the reviewer comments at the end of this letter.

As the corresponding author, your ORCID iD is verified in the submission system and will appear in the published article. PLOS supports the use of ORCID, and we encourage all coauthors to register for an ORCID iD and use it as well. Please encourage your coauthors to verify their ORCID iD within the submission system before final acceptance, as unverified ORCID iDs will not appear in the published article. Only the individual author can complete the verification step; PLOS staff cannot verify ORCID iDs on behalf of authors.

We look forward to receiving your revised manuscript.

Kind regards,

Dimitrios Vavylonis

Section Editor

PLOS Computational Biolog

Note: If the reviewer comments include a recommendation to cite specific previously published works, please review and evaluate these publications to determine whether they are relevant and should be cited. There is no requirement to cite these works unless the editor has indicated otherwise.

**Reviewers’ comments:**

Reviewer’s Responses to Questions

Reviewer #1: The authors are to be congratulated for a thorough update and response to reviews.

My only suggestion is that the authors make a stronger case that the non-sequential methods are still being used on the wet lab side. I had to look around to find the evidence, but indeed it does appear that this is the case. It would be nice to know that people have been using this more recently than the Choi 2022 paper.

Reviewer #2: The authors have successfully addressed my previous comments in the revision. I have no further concerns. Best, David Posada

**********

**Have the authors made all data and (if applicable) computational code underlying the findings in their manuscript fully available?**

The PLOS Data policy requires authors to make all data and code underlying the findings described in their manuscript fully available without restriction, with rare exception (please refer to the Data Availability Statement in the manuscript PDF file). The data and code should be provided as part of the manuscript or its supporting information, or deposited to a public repository. For example, in addition to summary statistics, the data points behind means, medians and variance measures should be available. If there are restrictions on publicly sharing data or code —e.g. participant privacy or use of data from a third party—those must be specified.

Reviewer #1: Yes

Reviewer #2: Yes

**********

PLOS authors have the option to publish the peer review history of their article (what does this mean?). If published, this will include your full peer review and any attached files.

If you choose “no”, your identity will remain anonymous but your review may still be made public.

**Do you want your identity to be public for this peer review?**    For information about this choice, including consent withdrawal, please see our Privacy Policy.

Reviewer #1: No

Reviewer #2: **Yes:**    David Posada

[NOTE: If reviewer comments were submitted as an attachment file, they will be attached to this email and accessible via the submission site. Please log into your account, locate the manuscript record, and check for the action link "View Attachments". If this link does not appear, there are no attachment files.]

**Figure resubmission:**

-->While revising your submission, we strongly recommend that you use PLOS’s NAAS tool (https://ngplosjournals.pagemajik.ai/artanalysis) to test your figure files. NAAS can convert your figure files to the TIFF file type and meet basic requirements (such as print size, resolution), or provide you with a report on issues that do not meet our requirements and that NAAS cannot fix.

After uploading your figures to PLOS’s NAAS tool - https://ngplosjournals.pagemajik.ai/artanalysis, NAAS will process the files provided and display the results in the "Uploaded Files" section of the page as the processing is complete. If the uploaded figures meet our requirements (or NAAS is able to fix the files to meet our requirements), the figure will be marked as "fixed" above. If NAAS is unable to fix the files, a red "failed" label will appear above. When NAAS has confirmed that the figure files meet our requirements, please download the file via the download option, and include these NAAS processed figure files when submitting your revised manuscript.

**Reproducibility:**

To enhance the reproducibility of your results, we recommend that authors of applicable studies deposit laboratory protocols in protocols.io, where a protocol can be assigned its own identifier (DOI) such that it can be cited independently in the future. Additionally, PLOS ONE offers an option to publish peer-reviewed clinical study protocols. Read more information on sharing protocols at https://plos.org/protocols?utm_medium=editorial-email&utm_source=authorletters&utm_campaign=protocols

# Author comment: Author response to Decision Letter 2

**DOI:** 10.1371/journal.pcbi.1014370.r004

25 May 2026

Attachment Submitted filename: Rebuttal letter - minor revision.pdf

# Editor report: Decision Letter 2

**DOI:** 10.1371/journal.pcbi.1014370.r005

**Authors:** Dimitrios Vavylonis

28 May 2026

Dear Ms. Pilarski,

We are pleased to inform you that your manuscript ‘Assessing the inference of single-cell phylogenies and population dynamics from CRISPR lineage recordings’ has been provisionally accepted for publication in PLOS Computational Biology.

Before your manuscript can be formally accepted you will need to complete some formatting changes, which you will receive in a follow up email. A member of our team will be in touch with a set of requests.

Please note that your manuscript will not be scheduled for publication until you have made the required changes, so a swift response is appreciated.

IMPORTANT: The editorial review process is now complete. PLOS will only permit corrections to spelling, formatting or significant scientific errors from this point onwards. Requests for major changes, or any which affect the scientific understanding of your work, will cause delays to the publication date of your manuscript.

Should you, your institution’s press office or the journal office choose to press release your paper, you will automatically be opted out of early publication. We ask that you notify us now if you or your institution is planning to press release the article. All press must be co-ordinated with PLOS.

Thank you again for supporting Open Access publishing; we are looking forward to publishing your work in PLOS Computational Biology.

Best regards,

Dimitrios Vavylonis

Section Editor

PLOS Computational Biology

***********************************************************

# Editor report: Acceptance letter

**DOI:** 10.1371/journal.pcbi.1014370.r006

**Authors:** Dimitrios Vavylonis

PCOMPBIOL-D-25-01344R2

Assessing the inference of single-cell phylogenies and population dynamics from CRISPR lineage recordings

Dear Dr Pilarski,

I am pleased to inform you that your manuscript has been formally accepted for publication in PLOS Computational Biology. Your manuscript is now with our production department and you will be notified of the publication date in due course.

The corresponding author will soon be receiving a typeset proof for review, to ensure errors have not been introduced during production. Please review the PDF proof of your manuscript carefully, as this is the last chance to correct any errors. Please note that major changes, or those which affect the scientific understanding of the work, will likely cause delays to the publication date of your manuscript.

Soon after your final files are uploaded, unless you have opted out, the early version of your manuscript will be published online. The date of the early version will be your article’s publication date. The final article will be published to the same URL, and all versions of the paper will be accessible to readers.

For Research, Software, and Methods articles, you will receive an invoice from PLOS for your publication fee after your manuscript has reached the completed accept phase. If you receive an email requesting payment before acceptance or for any other service, this may be a phishing scheme. Learn how to identify phishing emails and protect your accounts at https://explore.plos.org/phishing.

Thank you again for supporting PLOS Computational Biology and open-access publishing. We are looking forward to publishing your work!

With kind regards,

Judit Kozma

PLOS Computational Biology | Carlyle House, Carlyle Road, Cambridge CB4 3DN | United Kingdom ploscompbiol@plos.org | Phone +44 (0) 1223-442824 | ploscompbiol.org | @PLOSCompBiol
